# Severe Thrombocytopenia as the Main Manifestation of Childhood-Onset Systemic Lupus Erythematosus

**DOI:** 10.31138/mjr.33.4.459

**Published:** 2022-12-31

**Authors:** Cristian Quintana-Ortega, Agustín Remesal, Ana Pérez Vigara, Manuel Parrón-Pajares, Montserrat Bret, Rosa Alcobendas, Sara Murias

**Affiliations:** 1 Paediatric Rheumatology Department, La Paz Children’s Hospital, Madrid, Spain,; 2 Paediatric Radiology Department, La Paz Children’s Hospital, Madrid, Spain

**Keywords:** children, systemic lupus erythematosus, thrombocytopenia, intensive immunosuppression therapy

## Abstract

Thrombocytopenia is a common hematologic abnormality of childhood-onset systemic lupus erythematosus (cSLE). Although in most cases thrombocytopenia is mild, severe thrombocytopenia with bleeding complications might occur, and is further correlated with disease activity and a worse prognosis. We report two female patients with severe thrombocytopenia as the initial manifestation of cSLE, which were successfully treated by intensive immunosuppression including several high-dose methylprednisolone pulses and IV cyclophosphamide. Both patients were initially diagnosed with idiopathic thrombopenic purpura (ITP) refractory to conventional treatment and complicated with haemorrhagic manifestations. For this matter, patients with ITP should be assessed for the presence of ANA, anti-dsDNA antibodies, and complement levels, since they are at high risk to develop cSLE.

## INTRODUCTION

Childhood-onset systemic lupus erythematosus (cSLE) is a chronic multisystemic autoimmune disease characterised by the presence of autoantibodies secondary to immune dysregulation. cSLE represents approximately between 15 and 20% of all patients with SLE and can affect diverse organs of the body and might cause chronic inflammation.^1^ Therefore, it is a leading cause of morbidity and mortality. Pathogenesis of cSLE is not clearly understood; however, the combination of environmental, hormonal, and genetic factors is closely related to the susceptibility.^2,3^

Haematologic abnormalities, such as thrombocytopenia, haemolytic anaemia, and leukopenia, are common clinical features of cSLE. Prevalence of thrombocytopenia can range from 10% to 45% and may be the initial presentation in up to 15% of paediatric patients with LES^4^. Although thrombocytopenia in cSLE is usually mild (between 50x10^9 and 100x10^9), severe thrombocytopenia (less than 10x10^9) complicated with haemorrhagic manifestations might occur^5^.

Hereby, we described two paediatric female patients with severe thrombocytopenia as the initial manifestation of cSLE, further associated with haemorrhagic complications.

## CASE PRESENTATION

### Case 1

A 13-year-old female was admitted to our hospital with severe oral bleeding, epistaxis, and petechial rash. Initial blood cell count revealed severe thrombocytopenia (3x10^3^/μL), anaemia (6,2 g/dL) and lymphocytopenia (850/μL). She was diagnosed with idiopathic thrombopenic purpura (ITP) at local hospital five months before and oral prednisone at 1 mg/kg/day plus intravenous immunoglobulin at 1 g/kg (two doses) was started, without clinical and laboratory improvement.

The patient had a history of Raynaud′s phenomenon, alopecia, polyarthralgia, malar erythema, photosensitivity, and asthenia without weight loss. Twelve months before his admission, laboratory tests performed at local hospital revealed that the anti-nuclear antibodies (ANA) were positive (title 1/320) as well as anti-transglutaminase antibodies. She was referred to our department for a diagnosis.

**Figure 1.**
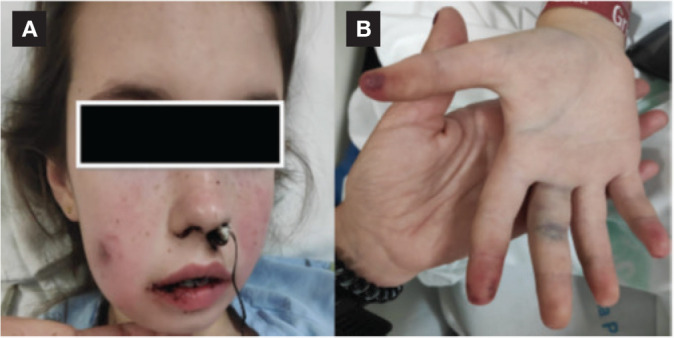
**A:** Malar erythema, epistaxis, and oral bleeding of Case I. **B:** Vasculitic-type cutaneous lesions on fingers.

On admission, she showed malar erythema, vasculitic-type cutaneous lesions on the fingers, and a purpuric rash on the feet and lower limbs (**Figure 1**).

Laboratory data indicated normal systemic inflammation markers. Haemolysis parameters (lactate dehydrogenase, haptoglobin, indirect bilirubin), renal function and urinalysis were within normal limits. Reticulocyte count was 3.5% and blood smear lacked schistocytes and abnormalities of leukocytes and erythrocytes. Analysis of plasma ADAMTS-13 activity revealed a normal value (73%). Microbiological tests for bacteria, virus (including cytomegalovirus, parvovirus B19, Epstein-Barr virus, herpes simplex virus) and fungus were negative. Immunological study was completed: positive direct antiglobulin (“Coombs”) test, positive ANA (title 1/1280), as well as anti dsDNA (36 IU / mL), anti-Ro/SS-A and anti-La antibodies. Furthermore, she presented hypocomplementemia (C3 73.30 mg/dL; C4 9.93 mg/dL).

Once the diagnosis of cSLE is considered, immunosuppressive therapy was intensified using methylprednisolone pulses (30 mg / kg / day) for 7 days, followed by oral prednisone (2 mg / kg / day), mycophenolate mofetil (600 mg/m^2^ twice a day) and hydroxychloroquine (200 mg twice a day).

**Figure 2.**
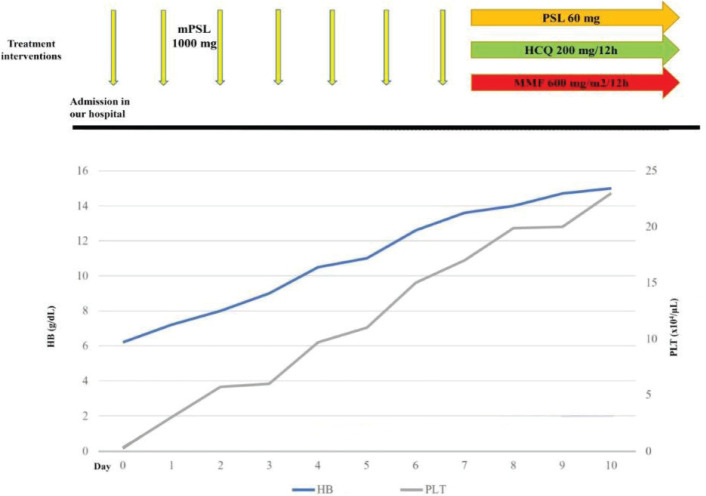
The clinical course of Case 1 after treatment initiation. The severe cytopenias and treatment interventions are shown. HCQ: hydroxychloroquine; mPSL: methylprednisolone pulses; MMF: mycophenolate mofetil; HB: hemoglobin; PLT: platelets

On day 7 after treatment initiation, she showed an improvement in laboratory parameters (haemoglobin 10.4 g/dl; platelet count 175x10^3^/μL) (**Figure 2**) and her symptoms gradually disappeared (resolution of haemorrhages, malar erythema, vasculitic-type cutaneous lesions and purpuric rash). In the following months the patient achieved a sustained haematological remission after tapering steroid therapy. Moreover, her serum anti-dsDNA antibody titre became negative and C4 recovered to normal levels.

### Case 2

**Figure 3.**
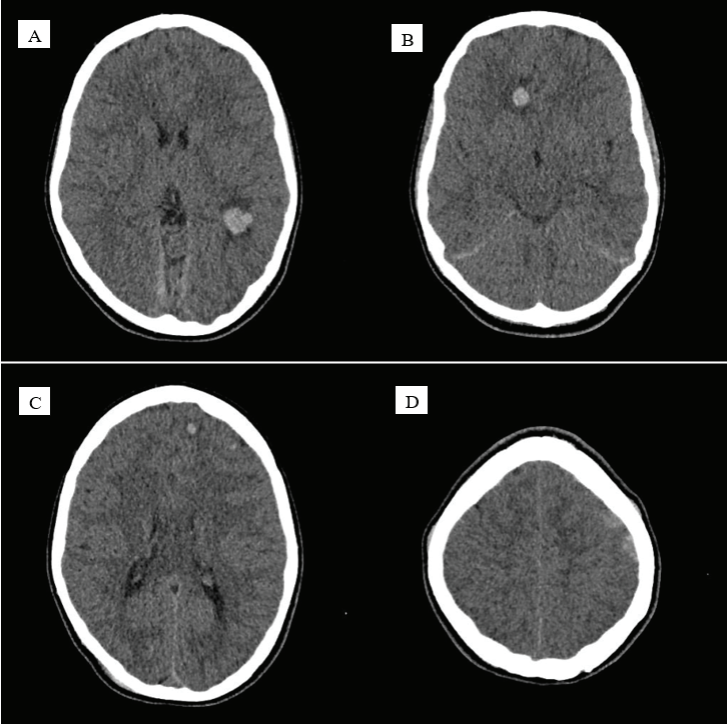
Head CT of Case 2 at admission showing multiple intracerebral haemorrhages in the left temporal-parietal lobe (A) and in both frontal lobes (B, C). Subdural hematoma in left parietal lobe is shown in picture D.

An 11-year-old female was admitted to our hospital with multiple intracerebral haemorrhages. She developed an intensive headache with photophobia and vomiting 10 days prior to her admission. At emergency department in our hospital, brain computed tomography (CT) showed multiple intracranial haemorrhages (**Figure 3**), and so she was referred to the Paediatric Intensive Care Unit for general monitoring and platelet transfusion.

She had a previous history of ITP (initial platelets: 4000/μL) diagnosed in our hospital two months before her admission. Prior immunological study revealed positive ANA (title 1/160) as well as triple positive for lupus anticoagulant, anti-cardiolipin, and anti-β2-glycoprotein I. Initially, she was treated with oral prednisone at 2 mg/kg/day plus intravenous immunoglobulin without laboratory and clinical improvement. She was referred to our department for a diagnosis.

On admission, physical exploration revealed purpuric rash, epistaxis and aphthous stomatitis. A malar erythema, Raynaud’s phenomenon, arthritis, and photosensitivity were not observed. The neurological examination showed right arm numbness and mild transient dysarthria. Blood cell count revealed severe thrombocytopenia (4x10^3^/μL), anaemia (8.9 g/dL) and lymphocytopenia (666/lL). Haemolysis parameters (lactate dehydrogenase, haptoglobin, indirect bilirubin), reticulocyte count (1.5%), renal function and urinalysis were within normal limits. Peripheral blood smear was performed, and no abnormalities of leukocytes and erythrocytes were found. Microbiological tests for bacteria, virus (including cytomegalovirus, parvovirus B19, Epstein-Barr virus, herpes simplex virus) and fungus were negative. Immunological study was completed: positive direct antiglobulin (“Coombs”) test, positive ANA (title 1/160), positive anti dsDNA (17 IU / mL) and a triple positive for lupus anticoagulant, anti-cardiolipin, and anti-β2-glycoprotein I (in two determinations separated by 12 weeks). No coagulation abnormalities and antiprothrombin antibodies were found. Complement values were: C3 85 mg/dL, C4 8,69 mg/dL.

**Figure 4.**
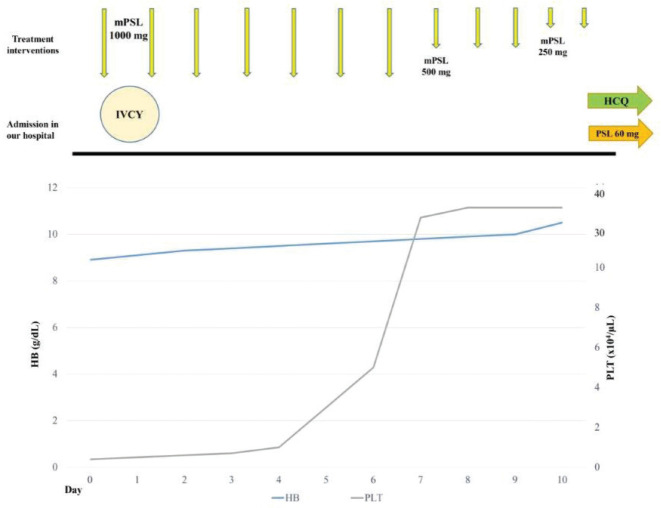
The clinical course of Case 2 after treatment initiation. The severe cytopenias and treatment interventions are shown. IVCY: intravenous cyclophosphamide; HCQ: hydroxychloroquine, mPSL: methylprednisolone pulses; MMF: mycophenolate mofetil, HB: haemoglobin; PLT: platelets

With the diagnostic suspicion of cSLE complicated with multiple intracerebral haemorrhages and severe thrombocytopenia, aggressive immunosuppressive therapy was initiated using a combination of high-dose methylprednisolone pulses (20 mg/kg/day for 7 days, 10 mg/kg/day for 2 days and 5 mg/kg/day for 2 days) and IV cyclophosphamide (500 mg/m^2^) followed by oral prednisone (2 mg / kg / day). On the 7th day after the beginning of the treatment a remarkable improvement was observed in laboratory parameters (platelets 450.000/μL, Hb 10.5 g/dL and lymphocytes 2380/μL) (**Figure 4**) with further disappearance of symptomatology (headache, vomiting, purpuric rash, epistaxis). The patient completed a treatment course of monthly IV pulse cyclophosphamide (500 mg/m^2^) for six months and achieved a sustained haematological remission after tapering steroid therapy. In addition, anti-dsDNA antibodies became negative and C4 recovered to normal levels.

## DISCUSSION

Haematological manifestations in cSLE have been reported to occur in up to 80% of cases^6^ and may be present at the beginning of the disease or develop during the course. Autoimmune thrombocytopenia is a common complication of childhood-onset lupus with a prevalence up to 25%^7^. It is usually mild, although it may be life-threatening in some cases due to haemorrhagic complications.^8^ In this regard, frequency of severe thrombocytopenia in children with SLE is approximately 13%^9^ and most common haemorrhagic manifestations in these patients included ecchymosis, epistaxis, and gingival bleeding; however, severe haemorrhagic complications such as gastrointestinal bleeding, intracranial haemorrhage, or diffuse alveolar haemorrhage may develop.^8^ Hereby, we described two childhood-onset systemic lupus erythematosus patients presenting with severe thrombocytopenia and haemorrhagic manifestations who were successfully treated with aggressive immunosuppressive therapy.

Differences in disease manifestations and disease severity between childhood-onset SLE and adult-onset SLE (aSLE) have been well established.^9^ In this way, recent meta-analysis performed by Livingston et al. confirmed higher average disease activity at diagnosis in cSLE than aSLE, as well as a higher prevalence of clinical manifestations such as fever, thrombocytopenia, haemolytic anaemia, mucocutaneous features, renal dysfunction, or nervous system involvement.^10^ Moreover, previous studies have reported a close association between thrombocytopenia and serious clinical manifestations in aSLE, such as neurological manifestations, kidney disorders, and haematological abnormalities.^5,11–14^ Consequently, severity of thrombocytopenia has been associated with an increase of the mortality rate. In this way, Jung et al. described a significantly higher mortality in those patients with aSLE and severe thrombocytopenia (14.9%) compared with patients with moderate and mild thrombocytopenia (8.8% and 0.8%, respectively).^15^

Due to all above-mentioned, and due to severity of the disease, urgency for response and unsuccessful first-line treatment in our two patients, we started aggressive immunosuppressive therapy consisted of several high-dose pulses of methylprednisolone (20–30 mg/kg/day; up to 7 doses) plus cyclophosphamide in Case 2 (because of nervous system involvement with intracerebral haemorrhages). On the 7^th^ day after treatment initiation, platelet count recovered within normal levels in both patients, besides improving anaemia and lymphopenia. Therefore, patients with severe thrombocytopenia and severe haemorrhagic manifestations at cSLE onset, should receive aggressive immunosuppressive treatment in order to achieve clinical remission, including several high-dose methylprednisolone pulses and/or IV cyclophosphamide. On admission, pancytopenia was observed in our two patients. The differential diagnosis of pancytopenia in SLE includes active SLE, secondary hemophagocytic syndrome/MAS, infection (in particular EBV, cytomegalovirus and herpes simplex virus), and myelosuppression secondary to antibody- or cytokine-mediated mechanisms. Negative microbiological tests would not support the infectious cause, and normal or high reticulocyte count found in both patients suggested that bone marrow suppression was unlikely, although a bone marrow aspiration was not performed to confirm it. Moreover, ferritin level, which has been considered as one important tool to differentiate MAS from active SLE,^16^ was normal in both patients, further accompanied by normal systemic inflammatory markers and triglycerides. Therefore, disease activity might be the principal cause of decrease in all three blood cell lineages. In addition, significant oral bleeding and epistaxis of Case 1 might contributed to the severe anaemia found at admission. In this regard, the high reticulocyte production rate, normocytic anaemia, normal ADMTS13 activity and the absence of haemolysis parameters could support the haemorrhagic aetiology of the profound anaemia.

Interestingly, lupus anticoagulant (LA) was positive in Case 2, who presented with intracerebral haemorrhages. When patients with LA presented with bleeding, a second coagulation abnormality should be considered such as an acute acquired hypoprothrombinaemia, which may develop as a result of the presence of anti-Factor II (FII) antibodies.^17^ Coagulation test in lupus anticoagulant-hypoprothrombinaemia syndrome (LA-HPS) revealed both prolonged activated partial thromboplastin (aPTT) and prothrombin time (PT), in combination with presence of LA. In this regard, Case 2 presented no coagulation abnormalities (normal aPTT, PT, and FII levels) and, additionally, no antiprothrombin antibodies were found. Therefore, bleeding disorder in this patient mainly be explained by severe thrombocytopenia.

Finally, our two patients were firstly diagnosed with ITP prior to their admission, which was refractory to conventional treatment. Several studies have assessed the possible role of ITP in early-stage systemic lupus.^18–22^ Thus, patients with ITP have a higher risk of SLE, estimating a prevalence ranging between 7% and 30% in patients with SLE.^23,24^ Nonetheless, the possible mechanisms regarding the association between ITP and the subsequent development of SLE remain unidentified. In this line, Zhu F-X et al. performed a retrospective cohort study to provide incidence of SLE in patients with ITP and the potential relationship between them.^25,26^ Using a nationwide population-based data to assess the risk of SLE in patients with ITP, they demonstrated that patients with ITP had 26 times higher risk of new-onset SLE compared with the control population. For this reason, a clinical follow-up of these patients with periodic assessment of ANA, anti-dsDNA antibodies and complement levels, may be necessary.^27^

In conclusion, severe thrombocytopenia with bleeding complications, although rare, might be the initial manifestation of childhood-onset SLE. Early diagnosis is necessary and intensive immunosuppressive treatment is required, since severe thrombocytopenia correlated with disease activity and predicted a worse prognosis. Paediatric patients with idiopathic thrombopenic purpura, especially those refractory to conventional therapy or complicated with haemorrhagic manifestations, should be assessed for the presence of ANA, anti-dsDNA antibodies and complement levels since they are at high risk to develop cSLE.
